# Cross-Sectional Profile Evolution of Cu-Ti Gradient Films on C17200 Cu by Vacuum Thermal Diffusion

**DOI:** 10.3390/ma15228002

**Published:** 2022-11-12

**Authors:** Yandan Zhu, Zecheng Li, Hongchao Bi, Qilong Shi, Yujun Han, Quanli Zhang

**Affiliations:** 1 Jiangsu Key Laboratory of Advanced Structural Materials and Application Technology, School of Materials Engineering, Nanjing Institute of Technology, Nanjing 211167, China; 2 Key Laboratory of Research on Hydraulic and Hydro-Power Equipment Surface Engineering Technology of Zhejiang Province, Hangzhou 310024, China; 3 College of Mechanical and Electrical Engineering, Nanjing University of Aeronautics and Astronautics, Nanjing 210016, China

**Keywords:** C17200 Cu alloy, Cu-Ti gradient film, cross-sectional profile, thermal diffusion

## Abstract

To improve the wear resistance and fatigue life of Cu alloys, surface modification by combining the magnetron sputtering of Ti film followed by vacuum thermal diffusion is always applied, where the structure and composition of the fabricated film play a determinant role on the mechanical properties. In the present work, the evolution of the layered structure and the element distribution of the formed multi-phases coating on C17200 Cu alloy are investigated by mathematical calculation based on Fick’s law, and the experimental verification by the thermal diffusion of the gradient Cu-Ti film was undertaken under different temperatures and durations. The results show that the layered structure of the fabricated coating is dependent on the Cu-Ti atom concentration, the increasing time and the temperature, where a single or stratified layer is formed due to the generated Cu-Ti intermetallics for the inter-diffusion between the Cu and Ti atoms. The atom distribution by the proposed simulation method based on Fick’s law corresponds to the experimental results, which can be applied to designing the structure of the modification layer.

## 1. Introduction

Cu alloys have been widely applied in electronics, electrics, aeronautics and astronautics. During the service of the Cu parts, the deficient wear resistance and the growth of the interface metallic compounds always result in an insufficient service life [1,2]. For example, bearings made of Cu alloys still require better surface mechanical properties to enhance the adhesion and bonding strength [3,4,5], and the controlled growth of the interface metallic compounds (IMC) in the microelectronic devices is now facing great challenges [6,7,8,9].

To improve the surface mechanical properties of the alloys, surface modification by combining magnetron sputtering and thermal diffusion process have been widely applied [10,11,12,13]. The hardness and the wear resistance of the coatings are closely related to the microstructure and the phase compositions, which not only depends on the process parameters but also the atomic ratio in the material [12,14,15]. The reaction between Cu and Ti atoms results in different Cu-Ti intermetallics [6,16], including CuTi, Cu_4_Ti, CuTi_2_, and so on [17], which provides certain improvement in wear resistance. In addition, the adhesion of the coating plays a critical role in the wear resistance [18], and a gradient Cu-Ti film is applied to fabricate a hard surface coating during the plasma nitriding process, which provides an intermediate layer for better support than the Cu substrate [14,18]. Moreover, vacuum annealing has been proposed as a simple and viable method to tune the diffusion process and the microstructure of the multi-phases coating [19]. To investigate the inter-diffusion of Cu and Ti atoms, the evolution of the cross-sectional profile is generally investigated by SEM imaging and EDS measurement, which both need to cut the sample, as the characteristics and evolution of the layered structure cannot be observed directly during the process [3,4,20]. Therefore, it is of great engineering importance to understand and master the interface formation process by a non-destructive method in vacuum thermal treatment [6,7,21]. However, only limited knowledge can be found on investigating the Cu-Ti systems by simulation and mathematical calculation.

In the present work, the simulation of atom diffusion and the evolution of the cross-section profile of the gradient Cu-Ti film on the C17200 Cu alloy surface is investigated by mathematic calculation and experiments, where the influences of the original Ti content in the Cu-Ti film, the diffusion time and the temperature are analyzed. The obtained results can provide certain instructions for the surface modification process and the predication of IMC growth in the brazing interface.

## 2. Materials and Experiments

The chemical composition of the used C17200 Cu material is shown in Table 1. The C17200 Cu substrates had a diameter of 20 mm and a thickness of 4.5 mm, which were firstly treated by water quenching after heating at 790 °C for 2 h for solid solution. The specimens were manually ground using silicon carbide papers from 240# to 2000# to achieve a fine finish, which was then polished and ultrasonically cleaned with acetone. The gradient Cu-Ti films were fabricated by the co-sputtering technique in a closed field unbalanced magnetron sputtering plating system (UDP-450/4, Teer Coatings Ltd., Droitwich Spa, UK), where the Cu target and the Ti target were simultaneously used in Ar plasma gas ambiance. The Cu-Ti films of different compositions were prepared on the surfaces of C17200 Cu substrates by adjusting the power of the Cu and Ti targets, as listed in Table 2, where the argon gas flow rate, the substrate bias and the deposition time were kept constant during the magnetron sputtering process, as listed in Table 3. The subsequent thermal diffusion of the obtained films was undertaken to generate a multi-phase coating in a vacuum resistance furnace at varying temperatures and time durations, where the samples were sealed into a vacuum tube before the thermal diffusion in a medium-temperature resistance furnace. Detailed information on the magnetron sputtering and thermal diffusion processes can be found in the previous studies [14,18].

The coated samples were then cut into two parts, and successive polishing of the cross-section by SiC abrasive papers was undertaken from 240# to 2000# to achieve a smooth morphology in the cross-section. The morphologies of the cross-section of the modified samples were then characterized by a SUPRA 55 SAPPHIRE scanning electron microscope (SEM), and the elemental distribution along the cross-sectional profile was analyzed using the equipped energy dispersive spectrometer (EDS). In the EDS measurement, the sampling grid points of the same span along the cross-sectional profile were selected. The composition and thickness of the pre-fabricated Cu-Ti films are listed in Table 4. It can be seen that the Cu-Ti films with Ti/Cu atomic ratios close to 7:1, 7:4, and 1:2 can be obtained by the three processes of F1, F2, and F3. 

The C17200 Cu alloy after magnetron sputtering is then subjected to vacuum thermal diffusion, the specific parameters of which are shown in Table 5.

## 3. Modeling and Calculation

### 3.1. Assumption and Basic Theory

The growth of the multi-phases coating is dependent on the concentrations of the Cu and Ti atoms, where the inter-diffusion of the atoms is of great significance. The thickness of the multi-phases coating is modeled by the mathematical calculation of Ti concentration along the cross-sectional profile based on Fick’s Law.

According to the previous diffusion experimental results of pure Ti film on C17200 Cu-Be and C61900 Cu-Al alloys [14,18,22], the coating is composed of the Ti-rich layer, the Cu-Ti intermetallic transition layer and the Cu-rich layer close to the matrix. The content of Ti drops with increasing depth. To simplify the model, the following assumption is proposed:

(1) Only the diffusion of Ti elements is considered as the titanium atoms diffuse faster than copper atoms in this alloy [23], and the influence of alloying elements is neglected;
(2) As the thickness of the multi-phase coating layer is much smaller than the sample during the Ti diffusion process, the process can be regarded as a diffusion problem in a one-dimensional and semi-infinite space;
(3) The internal temperature of the sample is uniform and constant during the thermal diffusion process;
(4) The Ti content in the Ti-rich layer changes linearly with time.

The atoms’ diffusion in a solid is divided into steady-state diffusion and unsteady-state diffusion. The steady-state diffusion can be described by Fick’s first law, where the atomic flux is proportional to the concentration gradient, and it can be expressed as follows:

(1) $$J=-D\frac{dc}{dx}$$

where *J* (kg/(m^3^·s)) is the diffusion flux, *D* (m^2^/s) is the diffusion coefficient, and *c* (kg/m^3^) is the mass concentration of the atoms.

For most situations, the atom concentration along the cross-sectional profile in the diffusion layer varies with time, and the diffusion dynamic is described by Fick’s Second Law. The concentration change at any point over time can be obtained by the difference between the inflow and outflow in a certain volume unit. The motion direction of the target atom is defined as the X-direction. Two parallel planes at a distance of d*x* perpendicular to the X-direction are selected, and the cross-sectional area is *A*. For the equilibrium relationship of substance, the cumulative rate in the unit volume (*V*) can be achieved as:

(2) $$V=V_{1}-V_{2}=J_{1}A-J_{2}A=-\frac{\partial J}{\partial x}Adx$$

where *V*_1_ (kg/(m·s)) is the inflow rate, *V*_2_ (kg/(m·s)) is the outflow rate, *J*_1_ (kg/(m^3^·s)) is the outflow flux, *J*_2_ (kg/(m^3^·s)) is the inflow flux, and *A* (m^2^) is the cross-sectional area. The matter accumulation rate in the unit volume can also be expressed as:

(3) $$V=\frac{\partial c}{\partial t}Adx$$

where *c* is the element concentration. Therefore, the following equation can be obtained:

(4) $$\frac{\partial c}{\partial t}Adx=-\frac{\partial J}{\partial x}Adx$$

That is,

(5) $$\frac{\partial c}{\partial t}=-\frac{\partial J}{\partial x}$$

Substituting Formula (1) into Equation (5), we can get:

(6) $$\frac{\partial c}{\partial t}=\frac{\partial }{\partial x}\left(D\frac{\partial c}{\partial x}\right)$$

Under the assumption that *D* is independent of concentration, the above equation can then be simplified as:

(7) $$\frac{\partial c}{\partial t}=D\frac{\partial^{2}c}{\partial x^{2}}$$

### 3.2. Mathematical Modeling and the Ti Atom Distribution in the Diffusion Process

**Figure 1:**
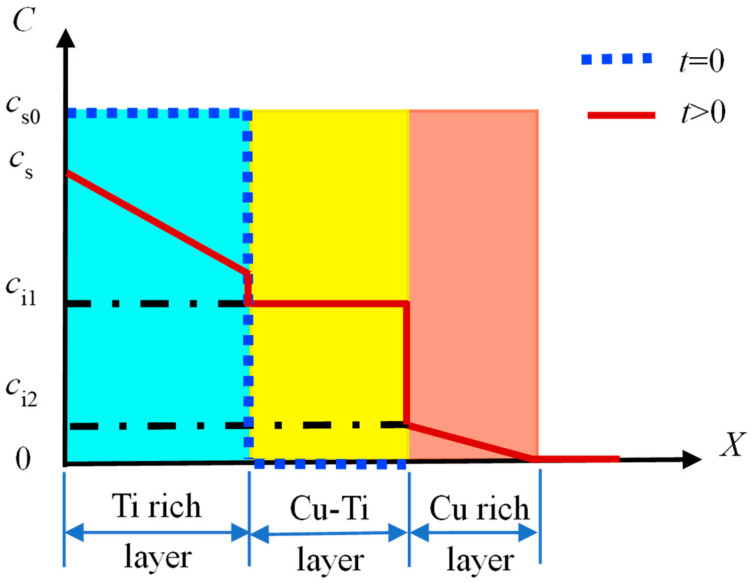
Schematic illustration of the cross-sectional profile for the multi-phase coating.

According to the physical model of the diffusion layer, the unsteady diffusion equation is solved through the initial conditions and the boundary conditions. The *X*-axis is perpendicular to the surface, which is the direction from the surface to the substrate. Taking the surface as the origin, the C–X coordinate is established. The schematic illustration of the cross-section structure of the modified layer is shown in Figure 1.

Before the diffusion of Ti atoms, the Ti concentration on the surface is *c_s_*_0_, and the concentration at the interface between the Ti-rich layer and the Cu-Ti reaction–diffusion layer is *c*_i1_. With the continuing process of diffusion, the Ti concentration (*c*_s_) on the surface decreases gradually. It was assumed that Ti concentration in the surface layer changed linearly:

(8) $$c_{s}=c_{s0}-Bt$$

where *c*_s0_ (kg/(m^3^·s)) is the original Ti concentration on the surface before diffusion, and B is a constant depending on the temperature.

The initial conditions and the boundary conditions can be expressed as Equations (9) and (10), respectively:

(9) $$\mathrm{When}\;t=0,\;c=c_{s0},x>0$$

(10) $$\mathrm{When}\;t>\;0,\;\{\begin{array}{c} \;c=c_{s},\;\;x=0\;\;\\ c=c_{i1},\;\;x=l_{1} \end{array}$$

where *l*_1_ is the distance of the interface between the Cu-rich layer and the Cu-Ti reaction–diffusion layer. Based on the above initial conditions and the boundary conditions, the solution of Equation (8), taking the error function, can be achieved as follows:

(11) $$c\left(x,t\right)=c_{s}-\left(c_{s}-c_{i1}\right)\mathrm{erf}\left(\frac{x}{2\sqrt{Dt}}\right)$$

**Figure 2:**
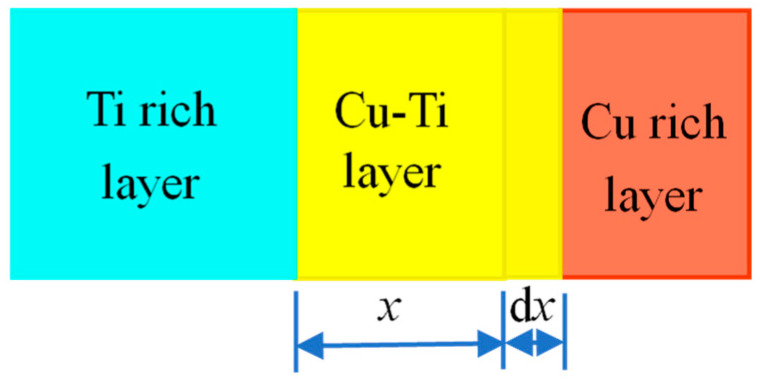
Schematic diagram of Cu-Ti reaction–diffusion layer without considering the Ti rich layer.

For the Cu/Ti reaction–diffusion layer, the Ti concentration is set as a constant value, *c*_i1_. The formation schematic of the Cu-Ti reaction–diffusion layer is shown in Figure 2. As Ti atoms continuously diffuse toward the Cu matrix, the thickness of the Cu-Ti reaction–diffusion layer grows. The solid-state reaction process is dependent on the diffusion speed.

The concentration at the interface of the Cu-Ti diffusion layer and the Cu-rich layer is *c*_i2_. After the duration of d*t*, the mass of Ti passing through the Cu-Ti diffusion layer is d*m*. According to Fick’s first law, the following relation can be built:

(12) $$\frac{dm}{dt}=-D{\left(\frac{dc}{dx}\right)}_{\xi =x}$$

Since the growth of the Cu-Ti compound layer is dependent on the Ti diffusion, the following equation can be obtained:

(13) $$dm=c_{i1}dx$$

If the diffusion is a steady process, we can get:

(14) $$-D{\left(\frac{dc}{dx}\right)}_{\xi =x}=D\left(\frac{c_{i1}-c_{i2}}{x}\right)$$

Combining Equations (12)–(14), we can derive:

(15) $$\frac{dx}{dt}=\frac{D\left(c_{i1}-c_{i2}\right)}{xc_{i1}}$$

By integrating both sides of Equation (15) and taking the boundary condition when *t* = 0 and *x* = 0, we can get:

(16) $$x^{2}=\frac{2D\left(c_{i1}-c_{i2}\right)}{c_{i1}}t$$

That is,

(17) $$x=\sqrt{\frac{2D\left(c_{i1}-c_{i2}\right)}{c_{i1}}t}$$

It can be found that the thickness of the Cu-Ti reaction–diffusion layer is proportional to the square root of time.

The diffusion model of the Ti element in the Cu-rich layer is a process with one end of the composition unaffected by diffusion. The initial conditions and the boundary conditions are as follows:

(18) When t=0, c=0,x>0 

(19) $$\mathrm{When}\;t>\;0,\;\{\begin{array}{c} \;c=c_{i2},\;\;x=0\\ c=0,\;\;x=\infty \end{array}$$

Based on the above initial conditions and boundary conditions, the solution of Equation (8), taking the error function into consideration, can be solved as follows:

(20) $$c\left(x,t\right)=c_{i2}\left(1-\mathrm{erf}\left(\frac{x}{2\sqrt{Dt}}\right)\right)\;$$

As far as the typical three-layered surface is concerned, the mathematical solution of the Ti concentration along the cross-sectional profile is achieved as follows:

(21) $$\{\begin{array}{c} c\left(x,t\right)=c_{s0}-Bt-\left(c_{s0}-Bt-c_{i1}\right)erf\left(\frac{x}{2\sqrt{Dt}}\right),x\in \left(0,l_{1}\right)\;\;\;\;\;\;\;\;\\ c\left(x,t\right)=c_{i1},x\in \left[l_{1},l_{1}+l_{2}\right]\;\;\;\;\;\;\;\;\;\;\;\;\;\;\;\;\;\;\;\;\;\;\;\;\;\;\;\;\;\;\;\;\;\\ c\left(x,t\right)=c_{i2}\left(1-\mathrm{erf}\left(\frac{x-l_{1}-l_{2}}{2\sqrt{Dt}}\right)\right),x\in \left(l_{1}+l_{2},\infty \right)\;\;\;\;\;\;\;\;\;\;\;\;\; \end{array}$$

Due to the time delay from the beginning of the diffusion to the formation of the compound interface layer, the growth duration of the compound layer can be modified. The correction coefficient ∆*t* is dependent on the concentration of Ti in the prefabricated film and the temperature. According to the law of mass conservation, the interface position of the Ti-rich layer and the intermediate Cu-Ti reactive diffusion layer can be determined.

### 3.3. Simulation of Ti Concentration Distribution in the Modified Layer

According to the preliminary experimental results of our research group, the content of Ti in the Cu-Ti reaction–diffusion layer is 30 at.%, and the corresponding mass concentration is 1.76 × 10^3^ kg/m^3^, namely, *c*_i1_ =1.76 × 10^3^ kg/m^3^. Four kinds of Cu-Ti film with different Ti contents between roughly 30 and 100 at.% are selected, which is also converted to mass concentration, namely, *c*_s0_, as listed in Table 6.

Combined with the solid solubility of Ti in Cu in the phase diagram, the Ti concentration (*c*_i2_) at the interface of the Cu-Ti diffusion layer and the Cu-rich layer at different temperatures was obtained, as listed in Table 7. The variation in the diffusion coefficient *D* with temperature can be obtained by the Arrhenius equation:

(22) $$D=D_{0}\exp\left(-\frac{Q}{RT}\right)$$

where D_0_ (m^2^/s) is the diffusion constant, R = 8.314 J/(mol·K) is the gas constant, *Q* (J) is the activation energy per mole, and *T* (K) is the absolute temperature. According to reference [23], the diffusion coefficient D_0_ for Ti in Cu is 0.693 × 10^−4^ m^2^/s, and the value of activation energy *Q* per mole is 1.96 × 10^5^ J/mol. The diffusion coefficients at different temperatures are shown in Table 7. 

**Figure 3:**
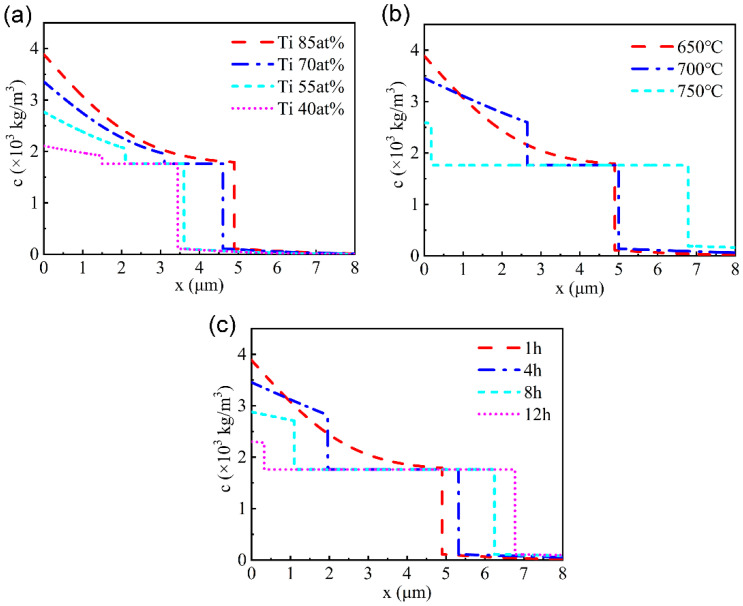
(**a**) The distribution of the Ti atoms along the cross-sectional profile after thermal diffusion at 650 ℃ for 1 h; (**b**) the distribution of the Ti atoms along the cross-sectional profile after thermal diffusion for 1 h at different temperatures; (**c**) the distribution of the Ti atoms along the cross-sectional profile after thermal diffusion at 650 ℃ for different durations.

According to the simulation results in MATLAB based on the above model and parameters, the distribution of Ti in the modified layer with different components was obtained after diffusion at 650 ℃ for 1 h, as shown in Figure 3a. The thickness of the modified layer is 3.5~5 μm, and the higher the Ti content in the prefabricated film, the greater the thickness of the modified layer after thermal diffusion.

The Ti atom distribution for the Cu-Ti film with 85 at.% Ti at 650 °C, 700 °C and 750 °C for 1 h was shown in Figure 3b. When the temperature rises to 700 °C, the total thickness of the modified layer grows and the thickness proportion of the Ti rich layer and the Cu-Ti reaction–diffusion layer also changes. When the temperature rises to 750 ℃, the surface layer almost transfers completely into the Cu-Ti reaction–diffusion layer. The distribution of Ti atoms for the Cu-Ti film with 85 at.% Ti after thermal diffusion for different time durations is shown in Figure 3c. The thickness of the modified layer gradually increases with the extension of time, and the thickness of the modified layer after diffusion at 650 ℃ for 12 h was nearly the same as that after diffusion at 750 ℃ for 1 h.

## 4. Experimental Verification

According to the simulation results of Ti atoms for the different fabricated Cu-Ti films and thermal diffusion parameters, three different Ti contents of 85 at.%, 65 at.% and 40 at.% in Cu-Ti films, as listed in Table 4, were selected for thermal diffusion at 650 ℃ and 750 ℃ for 1 h and 4 h, where the multi-phase coatings with different proportions of a 0–4 μm Ti-rich layer and a 1–7 μm Cu-Ti reactive diffusion layer are to be formed.

**Figure 4:**
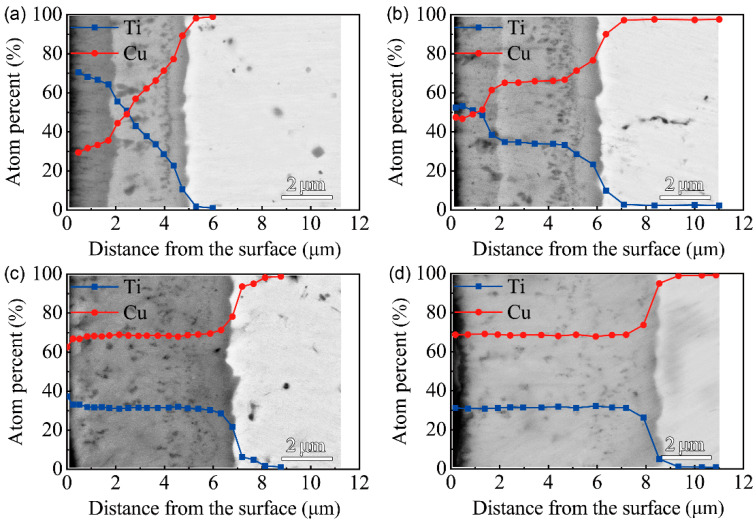
BSEM images (back-scattered electron image) of the cross-section and EDS results of F1 film on the C17200 Cu alloy after thermal diffusion: (**a**) 650 ℃ for 1 h; (**b**) 650 ℃ for 4 h; (**c**) 750 ℃ for 1 h; (**d**) 750 ℃ for 4 h.

The cross-sectional morphologies of the F1 film after vacuum thermal diffusion at different temperatures and time durations are shown in Figure 4. It can be seen that the multi-phase coatings mainly consist of two layers after thermal diffusion at 650 ℃, while the multi-phase coating is a uniform single layer after thermal diffusion at 750 ℃. The EDS results along the cross-section of the F1 film after thermal diffusion show that the inter-diffusion of Cu and Ti atoms occurs between the Cu-Ti film and the C17200 copper alloy matrix after treating for 1 h and 4 h at 650 °C. With the extension of the time, the Ti content on the surface drops; an intermediate layer with a Cu/Ti atom ratio of about 7:3 forms near the substrate, and the thickness of the intermediate layer gradually increases. From the EDS results along the cross-section of the multi-phases coating at 750 ℃ for 1 h and 4 h, a layer with a Cu/Ti atomic ratio close to 7:3 formed after the thermal diffusion for 1 h, as shown in Figure 4c,d. With the extension of the diffusion time, the thickness and composition of the multi-phases did not change obviously, indicating that the modified layer was relatively stable.

**Figure 5:**
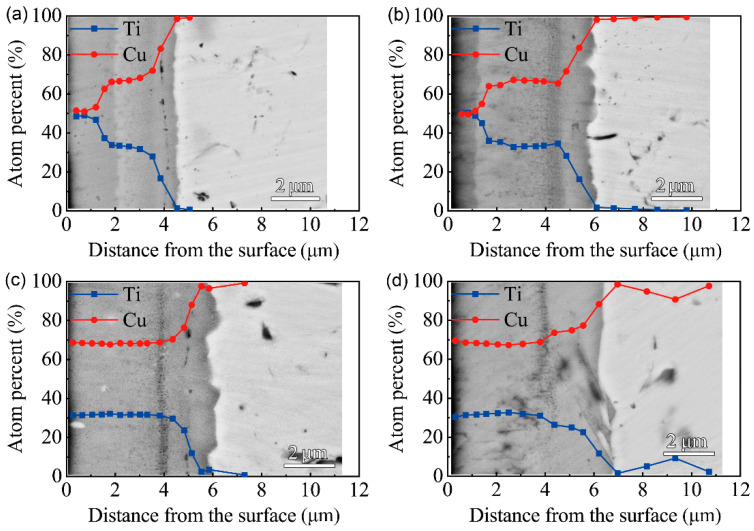
BSEM images of the cross-section and EDS results of F2 film on C17200 Cu alloy after thermal diffusion: (**a**) 650 ℃ for 1 h; (**b**) 650 ℃ for 4 h; (**c**) 750 ℃ for 1 h; (**d**) 750 ℃ for 4 h.

Figure 5 shows the BSEM images of the cross-section and EDS results of F2 film after vacuum thermal diffusion. Two layers of the multi-phases coatings formed at 650 ℃ for different time durations. With the extension of processing time, the thickness of the outer layer decreased, which indicates that the phase structure of the modified layer changed with the diffusion of Cu and Ti elements. After thermal diffusion at 750 ℃, a uniform modified layer formed. After 1 h and 4 h of vacuum thermal diffusion at 650 ℃, the inter-diffusion of Cu and Ti atoms resulted in a Cu/Ti atomic ratio of 1:1, with a thickness of about 1.5 μm. The ratio of Cu/Ti atoms in the intermediate layer reached 7:3, according to the EDS results, along the cross-section of the multi-phase coatings after thermal diffusion for 1 h and 4 h at 750 ℃. It can be seen that a modified layer with a Cu/Ti atomic ratio close to 7:3 formed after the thermal diffusion for 1 h due to the higher diffusion temperature. With the extension of the diffusing time, the thickness of the modified layer firstly increased and then stabilized at 7 μm, and the composition of the modified layer did not change significantly.

**Figure 6:**
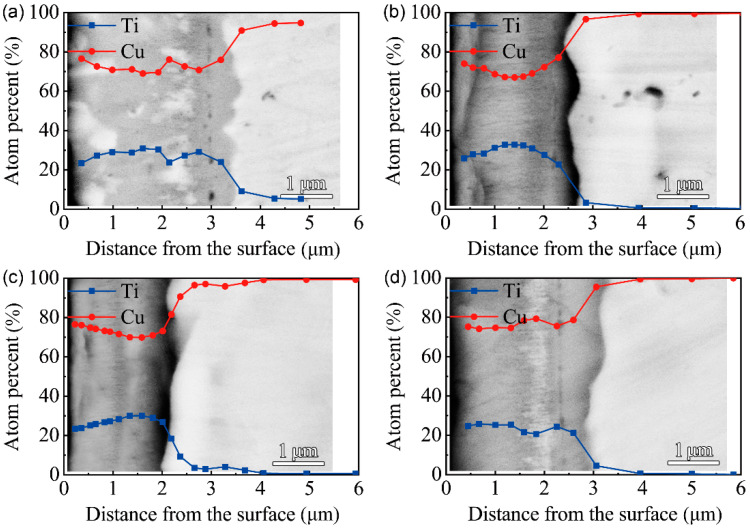
BSEM images of the cross-section and EDS results of F3 film on C17200 Cu alloy after thermal diffusion: (**a**) 650 ℃ for 1 h; (**b**) 650 ℃ for 4 h; (**c**) 750 ℃ for 1 h; (**d**) 750 ℃ for 4 h.

Figure 6 shows the BSEM images of the cross-section and EDS results of F3 film after vacuum thermal diffusion. There is no obvious stratification in the modified layer. After thermal diffusion at 650 ℃ for 1 h, the white block substances appeared near the surface. However, a banded white substance appeared near the base after thermal diffusion at 750 ℃ for 4 h. The EDS results along the cross-section indicate that the Cu/Ti atomic ratio of the modified layer of F3 film is close to 7:3. The Cu/Ti atomic ratio of the white substance in the cross-section is close to 4:1, which indicates that the white substance is the Cu-rich phase.

**Figure 7:**
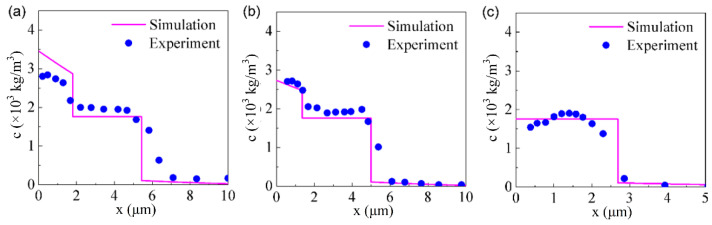
Comparison of the simulation and experimental results of Ti atoms along the cross-sectional profile after thermal diffusion at 650 ℃ for 4 h: (**a**) F1, (**b**) F2, (**c**) F3.

The Ti distribution along the cross-section profile of the multi-phases coatings has been compared with the simulation results at 650 ℃ for 4 h. The phase compositions of the C17200 Cu alloy and the pure copper are similar, as shown in Figure 7. It can be seen that the simulation results are in good agreement with the experimental results, which indicates that the diffusion model proposed in the present work can be applied to predict the concentration distribution of Ti and the thickness of the modified layer under the given parameters, and thus guide the experimental research.

## 5. Conclusions

The evolution of the layered structure and the element distribution of the multi-phase coating formed on the C17200 Cu alloy in thermal diffusion has been investigated based on the Fick’s law, and the experimental verification was undertaken based on the SEM imaging and the EDS measurement of the element distribution along the cross-section. The evolution of the layered multi-phase coating is dependent on the original atomic concentration of the fabricated Cu-Ti film, the increasing time and the temperature, where a single or stratified layer is generated due to the formation of the Cu-Ti intermetallics for the inter-diffusion between the Cu and Ti atoms. The atom distribution by the proposed simulation method based on Fick’s law achieves good consistence with the experimental results, which can be applied to design the structure of the modification layer of Cu alloys and predict the evolution of the IMC in the service stage at high temperatures.

## Figures and Tables

**Table 1:** The chemical composition of C17200 Cu alloy (wt. %).

Be	Co	Ni	Fe	Si	Cu
1.96	0.10	0.12	0.15	0.12	Bal.

**Table 2:** The target powers for fabricating films with different Cu and Ti contents by magnetron sputtering.

Process	Ti Target Power (W)	Ti Target Current (A)	Cu Target Power (W)	Cu Target Current (A)
F1	2500	7.00	100	0.33
F2	2000	5.69	300	0.84
F3	1000	3.02	450	1.18

**Table 3:** The fixed parameters for the magnetron sputtering process.

Total Ar Pressure (Torr)	Substrate Bias (V)	Sputtering Time (h)	Substrate-to-Target Distance (mm)	Workpiece Rotation Speed (r/min)	Vacuum Chamber Temperature (°C)
3.7 × 10^−2^	−70	3	120	5	20~150

**Table 4:** The composition and thickness of the fabricated Cu-Ti films formed by magnetron sputtering on C17200 Cu alloys.

Materials	Coating Process	Film Composition		Film Thickness (μm)
		Cu (at.%)	Ti (at.%)	
C17200	F1	13	87	3.0
	F2	37	63	3.0
	F3	67	33	2.5

**Table 5:** The thermal diffusion parameters for the Cu-Ti films on the C17200 Cu alloys.

Temperature (°C)	Duration (h)	Coating Process	Substrate Material
650	1	F1 F2 F3	C17200
	4		
750	1	F1 F2 F3	C17200
	4		

**Table 6:** The concentration of Ti atoms in the designed coating.

Ti content (at.%)	*c*_s0_ (×10^3^ kg/m^3^)
85	4.03
70	3.50
55	2.91
40	2.25

**Table 7:** The concentration of Ti (*c*_i2_) and the diffusion coefficient at different temperature.

Temperature (°C)	*c*_i2_ (×10^3^ kg/m^3^)	*D* (×10^−15^ m^2^/s)
650	0.11	0.56
700	0.14	2.08
750	0.19	6.80

## Data Availability

The data presented in this study are available on request from the corresponding author.
